# The Impact of Ethnicity on the Response to Eltrombopag in Patients With Immune Thrombocytopenia (ITP) in Qatar: A Single Institution Experience

**DOI:** 10.7759/cureus.25701

**Published:** 2022-06-06

**Authors:** Mohamed A Yassin, Rola Ghasoub, Ashraf Soliman, Omar Ismail, Abdulqadir J Nashwan, Awni Alshurafa, Firdous Ghori, Deena Sideeg, Anas Hamad, Radwa Hussein, Randa Al-Okka, Prem Chandra, Aya Alasmar

**Affiliations:** 1 Hematology, National Centre for Cancer Care and Research, Hamad Medical Corporation, Doha, QAT; 2 Pharmacy, Hamad Medical Corporation, Doha, QAT; 3 Pediatric Endocrinology, Hamad Medical Corporation, Doha, QAT; 4 Hematology and Medical Oncology, Hamad Medical Corporation, Doha, QAT; 5 Oncology, Qatar University, Doha, QAT; 6 Nursing, University of Calgary In Qatar, Doha, QAT; 7 Nursing Education, Hamad Medical Corporation, Doha, QAT; 8 Medical Research Center, Hamad Medical Corporation, Doha, QAT

**Keywords:** thrombocytopenia, tpo agonists, ethnicity, eltrombopag, immune thrombotic purpura

## Abstract

Background: Eltrombopag olamine (ELT) is a synthetic nonpeptide with a low molecular weight that has been investigated in various phase-3 studies and shown to be efficacious at a typical dose of 50 mg. Varied ethnic groups have reported different responses to ELT.

Aim: The aim is to examine the efficacy of ELT in Asian and Arab patients with immune thrombocytopenia (ITP) from the Indian subcontinent by starting with (12.5 mg, as a minimum dose) and gradually increasing to a maximum dose of 50 mg.

Methods: Between January 2015 and January 2019, we reviewed the electronic health records of non-Arab Asian (n = 17) versus Arab (n = 41) patients who were ≥18 years old, residing in Qatar, and with confirmed diagnoses with chronic ITP and under active treatment with a platelet count of 30,000/L, and bleeding symptoms. Following receiving ELT for three months or longer at various dosages, patients' response was examined.

Results: After three months of ELT therapy, the response rate (platelet count of 50,000/L) was equivalent in non-Arab (88.2%) versus Arab (87.5%) patients. However, to achieve an adequate response, 26% of Arab patients required a lower dose of 12.5 or 25 mg, and 41.5% required a higher dose of 50 mg.

Conclusion: In adult chronic ITP patients, ELT is typically well-tolerated and delivers the desired outcomes. In 67.5% of Arab patients, smaller dosages of ELT (12.5-50 mg) were helpful in sustaining acceptable PLT levels. This helps patients get the most benefit at the lowest feasible dose, reducing toxicity and expense.

## Introduction

Eltrombopag is a low molecular weight, synthetic non-peptide biphenyl hydrazone that works by selectively binding to and activating the thrombopoietin receptor causing a cascade of intracellular signal transduction including conformational change of the receptor and activation of JAK/STAT signaling pathway resulting in increased proliferation and differentiation of megakaryocytes. Eltrombopag half-life elimination is around 21 to 32 hours in healthy individuals while it is 26 to 35 hours in patients with immune thrombocytopenia (ITP) [1]. There are five different Thrombopoietin receptor agonists (TPO-RA): avatrombopag, lusutrombopag, eltrombopag, romiplostim, and recombinant human thrombopoietin (rhTPO) [2]. Though all agents are TPO-RA there are variations among them. In our facility, we have two available agents (eltrombopag and romiplostim). Whilst eltrombopag acts at the transmembrane site, romiplostim works by directly and competitively binding to the TPO receptor binding site. Romiplostim appears to mainly stimulate mature precursors, eltrombopag acts earlier in the pathway by stimulating Megakaryocyte precursor cells and megakaryocytes differentiation [3]. Recent clinical trials are unraveling a new mechanism of action in which eltrombopag is involved in the immunoregulation of T-regulatory cells (TREGs). It has been noticed that ITP patients have an increased TREGs activity post eltrombopag initiation, this resulted in reduced production of IL-2 and increased levels of TGF-b signifying that eltrombopag enhanced Treg function and restored immune tolerance. In ITP, the phagocytic hyperactivation involves both FcγRI and FcγRIIa, while FcγRIIb exhibits an inhibitory role. In recent studies, eltrombopag is thought to restore the balance of the Fc-γ receptor (FcγR) toward the FcγRIIb inhibitory role. In vitro phagocytosis experiments confirmed these findings, revealing a decrease in monocyte/macrophage phagocytic ability and more evidence that eltrombopag has an immunomodulatory impact on ITP [4,5].

Platelet formation is stimulated by TPO-RA. Rather than achieving a precise platelet count, the objective of therapy for individuals with chronic ITP is to lower the risk of severe bleeding [6,7]. Treatment modalities for patients with chronic ITP are mostly focused on minimizing immune-mediated platelet breakdown (e.g., immunosuppressive drugs, intravenous immunoglobulin, corticosteroids, and splenectomy) [8]. Nevertheless, a considerable proportion of patients with ITP, even those who have had a splenectomy, develop resistance to these treatments [9-11]. The efficacy of eltrombopag was investigated in a double-blind, phase 3, placebo-controlled trial of previously treated patients with ITP (RAISE) [10]. Compared to placebo, eltrombopag treatment exhibited response in 79% of patients (response defined as a platelet count of 50,000-400,000 per μL). And the long-term study showed that after three years of treatment; eltrombopag sustained long-term efficacy and safety with dose adjustment according to the response [12,13]. It has been also studied in multiple different populations including those of Japanese and Korean descent. However, eltrombopag effects have not been previously studied in the Arab and East Asian populations. In this study, we attempt to evaluate the role of ethnicity (Arab versus Indian sub-continent patients), if any, in response to eltrombopag among patients in Qatar. Additionally, evaluate the efficacy of different doses in these two ethnic groups (100, 75, 50, 25, 12.5 mg).

## Materials and methods

Medical data from January 2015 to January 2019 were reviewed in retrospective research. Patients had to be at least 18 years old, have had ITP for at least six months, have a baseline platelet count of less than 30 ×10^9^/L, and have been resistant to one or more prior ITP therapy. The eligibility criteria were based on the RAISE study, with minor changes [10]. Patients of Arab or Indian subcontinent ancestry who were taking concurrent ITP drugs were eligible provided their dosages had been stable for at least four weeks prior to the trial.

The exclusion criteria included: 1) Treatment for Helicobacter pylori eradication has to be performed at least three months before to enrollment. 2) having HIV, hepatitis B or C infections, 3) any serious medical illness or blood problem other than ITP or platelet aggregation irregularity, 4) a history of thrombosis (arterial or venous) within the previous year, or a malignancy.

Patients’ response was evaluated using two parameters:

1. Clinical parameter where patients were classified as No bleeding (complete response), cutaneous bleeding (partial response), mucocutaneous bleeding (no response), and major bleeding (Central Nervous System/Gastrointestinal/genitourinary (no response).

2. Laboratory parameters by evaluating platelets number and mean platelets volume (MPV) before and after treatment (Table1).

**Table 1:** Response assessment: response time and quality CR: complete response, R: Response, NR, Non-Response [14]

CR	Platelet count of 100 x 109/L with no bleeding.
R	Platelet count of 30 x 10^9^/L, with a 2-fold rise in baseline count and no hemorrhage
Period to response	the time between commencing therapy and achieving CR or R.
NR	platelet count less than 30 X 109/L or hemorrhage with a 2-fold rise in baseline platelet count

The study was carried out with the agreement of the Medical Research Center (MRC-01-19-159) and in strict accordance with the principles of the “Declaration of Helsinki,” Good Clinical Practice (GCP), and Qatari laws and regulations.

Statistical analysis

Categorical data were summarized and expressed as frequency (percentage). Continuous/Quantitative data were expressed as mean standard deviation if normally distributed or median (inter-quartile range [IQR]) if not. Associations between two or more qualitative variables (gender, ethnicity, treatment response, etc.) were assessed using Pearson Chi-square and Fisher Exact tests, as appropriate. Quantitative data such as age, BMI, WBC, platelet count, and hemoglobin levels among the two independent groups were analyzed using unpaired “t” test or Mann-Whitney U tests, as appropriate. Univariate logistic regression was used to analyze the relationship between all potential predictor variables. A two-sided P-value < 0.05 will be considered statistically significant. All statistical analyses will be done using statistical packages SPSS 22.0 (SPSS Inc., Chicago, IL) and Excel statistical Pack 2010.

## Results

A total of 58 patients were identified. Major demographic findings should be stated briefly. More details are shown in Tables 2-5.

**Table 2:** Baseline characteristics of enrolled ITP patients Chi-square Fisher Exact test was used for 2x2 tables and for tables more than 2x2, Yates corrected Chi-square test was applied in the case of small cell frequencies (50% or more cells have expected frequencies <5), whereas quantitative outcome measures were compared by using an unpaired t-test to compute respective statistical P-values. All percentages were computed using non-missing data. ELT - Eltrombopag olamine

	Arabs N=41 (70.7%)	Non -Arab Asians N=17 (29.3%)	P-value*
Age (years)	39.46±17.70	40.47±13.49	0.834
Gender			
Male	15 (36.6%)	13 (76.5%)	0.006
Female	26 (63.4%)	4 (23.5%)	
Body weight (Kg)	79.32±20.51	68.11±12.63	0.041
ELT dose (mg)			
12.5 mg	2 (4.9%)	0 (0)	0.942
25 mg	9 (22%)	2 (11.8%)	
50 mg	17 (41.4%)	9 (52.9%)	
75 mg	13 (31.7%)	6 (35.3%)	
Splenectomy Status			
Yes	8 (19.5%)	1 (5.9%)	0.192
No	33 (80.5%)	16 (94.1%)	
Prior Therapies for ITP			
Corticosteroids	33 (80.5%)	12 (70.6%)	0.411
Rituximab	15 (36.6%)	4 (23.5%)	0.335
IVIG	33 (80.5%)	13 (76.5%)	0.731
Others			
Romiplostim	4 (9.8%)	2 (11.8%)	0.808
Azathioprine	2 (4.9%)	0 (0%)	--

 

**Table 3:** Hematological parameters, dose of eltrombopag and % response in Arabs versus Non-Arabs *Chi-square and unpaired t-tests were applied to compute the respective statistical P-values. **Complete or partial responses were computed using response criteria stated in section method (Table 5).

		Age at Diagnosis (years)	Platelet at Diagnosis (x 1,000)	Hb at Diagnosis	WBC at Diagnosis	Eltrombopag dose (mg)	% Complete or partial response
Arabs	Mean	33.15	25.32	12.31	7.94	50.61	36/40 (90.0%)
n = 41	SD	19.86	25.39	2.18	2.63	20.34	
Non -Arab Asians	Mean	36.47	26.06	14.14	9.89	55.88	
n = 17	SD	10.98	24.90	2.04	5.42	16.61	15/17 (88.3%)
P- value*		0.420	0.919	0.004	0.173	0.349	0.843

 

**Table 4:** Percent response of Arab vs non-Arab patients to eltrombopag *significant when p<0.05

RESPONSE:	PLT :1-30 (x 1,000)	PLT: >31-50 (x 1,000)	PLT >51-100 (x 1,000)	PLT >100 (x 1,000)	P-value*
Non-Arab Asians (n = 17)	2 (11.8%)	2 (11.8%)	1 (5.9%)	12 (70.6%)	0.235
Arabs (n = 41)	4 (10%)	5 (12.50%)	12 (30%)	19 (47.5%)	
Total (n= 58)	6 (10.5%)	7 (12.3%)	13 (17.45%)	31 (54.4%)	

**Table 5:** Percent response to different doses in Arabs and Non-Arab Asians *significant when p<0.05

Dose Eltrombopag	12.5 mg	25mg	50 mg	75 mg
Arabs (n=41)	4.9%	22.0%	41.5%	31.70%
Non-Arabs Asians (n = 17)	0.0%	11.8%	52.9%	35.3%
P value	0.56	0.42	0.43	0.71

Approximately 70.5% of non-Arab Asians and 46.3% of Arabs achieved CR on an average dose of 50 mg daily of eltrombopag olamine (ELT). In a more clinical sense, acceptable response or remission is also defined as platelets count of > 30,000 or at least twofold increase in the baseline count and absence of bleeding. Almost 90% of our patients achieved at least acceptable remission. In terms of response to lower dosing, 22.41% (13/58) of our patients responded to lower dosing of eltrombopag (84.6% of which are Arabs with 72.7% of which were females). More than 26% of Arab patients responded to doses of less than 50 mg (22% [25 mg/d] and 4.9% [12.5 mg/d]) whereas 17.6% of Asians achieved response on dosing of 25 mg/d. Only 22% of Arab patients and 35.3% of Non-Arab Asian patients required 75 mg or more of eltrombopag to achieve acceptable control.

A sub-analysis revealed that 70% of Arab patients who obtained CR (14/20) were females, whereas 33.3% (3/9) were Asian females. Females made up two-thirds of Arab patients who achieved clinical remission, whereas men made up more than two-thirds of non-Arab Asian patients. Most patients in both ethnic groups were diagnosed incidentally or presented with minor bleeding (i.e., ecchymosis, epistaxis or gum bleeding). Only two patients in each group presented with major bleeding episode such as subdural hemorrhage. The majority of patients (75%; 3/4) who presented with major bleeding required higher dosing of eltrombopag (75 mg); only one patient did not achieve remission with ELT. Nine of our patients were splenectomized. The majority of splenectomized patients managed to achieve acceptable response with an average dose of 51.3 mg of ELT.

## Discussion

The safety and efficacy of eltrombopag have been studied in multiple trials in the past. Based on this data, the dosing of eltrombopag was established at 50 mg once daily for most patients: with a maximum daily dose of 75 mg [15].

ELT’s efficacy has been studied in phase 3, a double-blind, placebo-controlled study of previously treated ITP patients (RAISE) [10]. Compared to placebo, eltrombopag treatment exhibited optimal response in 79% of patients. And the long-term study showed that eltrombopag sustained long-term efficacy and safety after three years of treatment [12,13]. Nonetheless, interethnic differences in the pharmacokinetics were confirmed by Tomiyama et al. in multicenter research, 23 Japanese patients with previously treated chronic ITP were evaluated in a six-week randomized, double-blind, placebo-controlled phase (15 eltrombopag and eight placebo) and a six-month open-label phase (23 eltrombopag). Twenty-two percent (5/23) of patients reacted to 12.5 mg of ELT in the first three weeks of therapy [16,17]. This study showed that the starting daily dose of 12.5 mg is recommended for Japanese ITP patients [16,17]. This has been the first study in Asia, where the interethnic traits showed an effect on the dosing of the drug. However, it only studied the Japanese population. Our study is expanding more on the concept of possible (interethnic) differences in response to ELT. The Arab world and the Indian sub-continent patients’ responses to different dosing of eltrombopag have never been studied. This study is the first in the region to evaluate patients’ response in Qatar to the different dosing of ELT. Similar to the study by Tomiyama et al. [16], more than half (35/58) of our patients achieved complete remission (platelet count of >100 x 10^3^/L). The response rate to eltrombopag after three months of treatment was comparable among the two different ethnic groups (Arabs 87.5% and non-Arab Asians 88.2%). Additionally, a good number of patients achieved clinical response with lower dosing of eltrombopag (as low as 12.5 mg), and most were maintained on 50 mg daily dosing). Only a minority of patients required higher dosing of 75 mg (10%).

The area under the curve (AUC) was nearly two-fold higher among Japanese healthy volunteers than among non-Asian volunteers after exposure to eltrombopag, and around 87% higher among patients of East Asian origin than those of non-East Asian descent [10]. A recent Korean trial investigated the required eltrombopag dose to achieve and maintain a safe platelet count in adult refractory immune thrombocytopenia. In that study, low-dose eltrombopag effectively maintained the target platelet count in almost 66% (12/18) of patients [6].

Many variables impact drug response, including genetic variation in the enzymes and transporters that metabolize the drug. Because the frequency varies greatly between ethnic groups, it has an influence on the proper selection and dose of various medications in different populations.

The most common adverse events reported with the use of eltrombopag include abnormal hepatic function tests (transaminitis) and thromboembolic events. The incidence of AEs was 73% (11/15) for patients receiving eltrombopag vs. 25% (2/8) for patients receiving placebo in the study by Tomiyma et al. However, most of these incidences were mild to moderate in severity [17]. We had no thromboembolic events or cases of transaminitis. This could be attributed to the use of lower doses of the drug. This is an exploratory study that needs to be confirmed with a large-scale study to validate our results, also a detailed pharmacokinetic profiling of eltrombopag in Arab and Indian sub-continent population which could explain the response to low doses of eltrombopag need to be explored.

## Conclusions

In conclusion, eltrombopag is well-tolerated in adult ITP patients and successfully reaches target platelet counts. Low dosages of eltrombopag were shown to maintain safe platelet levels. These findings showed that the lowest feasible dose can provide the greatest benefit to the patient and that this approach can decrease or eliminate adverse effects. Treatment recommendations that are customized to ethnic variations can help individuals and the healthcare system save money.
